# Supplementary material for: Role of Electron Spin, Chirality, and Charge Dynamics in Promoting the Persistence of Nascent Nucleic Acid–Peptide Complexes
Source: J Phys Chem B. 2025 Apr 15;129(16):3978–87. doi: 10.1021/acs.jpcb.5c01150 (PMC12035798; doi:10.1021/acs.jpcb.5c01150)
Supplement: Supplementary file 1 — jp5c01150_si_001.pdf [file jp5c01150_si_001.pdf]

Supplementary Materials for

The Role of Electron Spin, Chirality, and Charge Dynamics in Promoting the Persistence of Nascent Nucleic Acid-Peptide Complexes

Pratik Vyas[1†#], Kakali Santra[1†], Naupada Preeyanka[1], Anu Gupta[1], Orit Weil-Ktorza[2], Qirong Zhu[1], Norman Metanis,[2] Jonas Fransson,[3] Liam M. Longo[4,5] and Ron Naaman[1*]*

[†]These authors contributed equally to this work.

[1]Department of Chemical and Biological Physics, Weizmann Institute of Science, Rehovot, 76100, Israel
[2]Institute of Chemistry, The Hebrew University of Jerusalem, Jerusalem 9190401, Israel
[3]Department of Physics and Astronomy, Uppsala University, Uppsala 752 36, Sweden
[4]Earth-Life Science Institute, Institute of Science Tokyo, Tokyo 152-8550, Japan
[5]Blue Marble Space Institute of Science, Seattle, Washington 98104, USA
[#]Current address: Department of Chemistry, University of Basel, Switzerland

***Corresponding Authors**

Ron Naaman: ron.naaman@weizmann.ac.il;
Pratik Vyas: pratikbharat.vyas@unibas.ch

This PDF file includes:

Supplementary Text: Quantum mechanical simulations
Supplementary Text: Materials and Methods
Supplementary Figures: S1 to S10
Supplementary Tables S1 to S4
References (1 to 5)

Supplementary Text

Quantum mechanical simulations

For testing if the proposed mechanism of spin-dependent charge reorganization shown in Figure 5 (main text) can in principle take place, we performed simplified quantum simulations to model the dynamics of the charge density with a model system, comprising of a chain of $\mathbb{M}$ sites distributed in helical (chiral) or a zig-zag (achiral) shape (**Figure S10, panel A**). We shall refer to these model systems as the chiral and achiral molecules. Each site may hold an electron at the energy ε_m and is coupled to its nearest and next-nearest neighbor by both elastic (t_0, λ_0) and inelastic (t_1, λ_1) components, where the inelastic components account for nuclear vibrations that are coupled to the electronic charge (t_1) and to the electronic spin through the vibrationally enhanced spin-orbit coupling (λ_1). The chiral or achiral structures are controlled by the next-nearest neighbor interaction. The effect of the perturbing charge on the chiral system is provided by an additional external electron, illustrated by the magenta ball in **Figure S10, panel A**, where the green line between this charge and the system signifies the (time-varying) electrostatic coupling between the DNA and the protein. The details of setting up the molecule are described later in **Materials and Methods** (*Simulation Methododogy*) and in Refs. 1,2.

The simulations of the charge dynamics in the chiral and achiral chain are undertaken by varying the coupling between the external charge and the system harmonically as $U_0(1 + \cos v_0 t)$, where U_0 defines the maximum coupling strength, v_0 the frequency of the variations, and t is the time. The method used for the simulations of the dynamics is described in the Supplementary Information and in Ref. 2.

In Figure S10, panel B, we have plotted the density of electron states for the chiral and achiral molecules. The spectrum, which is confined below the chemical potential, zero vertical line, should be considered as the set of occupied orbitals whereas the unoccupied orbitals are assumed to be located at energies beyond 4 eV. It should be noticed that the densities for the two molecules are identical.

By the time-dependent variations in the local charge conditions near one of the edges of the system that models the molecule (protein), the internal charge of the molecules forced to react in response to these changes. As a measure of the variations, we calculate the electric polarization vector $P = (Px, Py, Pz)$ of the system with respect to its center of mass. In both types of molecules, there is a

distinctive time-dependence imposed on the charge polarization (**Figure S10C** and **S10D**), where largest vector component of P is plotted as function of time. Here, we compare the dynamical responses for frequencies between 1 MHz and 10 THz, and the responses are plotted on the same unit less time axis to enable a comparison.

First, one can notice that the overall polarization is stronger in the chiral than in the achiral structure, both in amplitude and mean value. Second, it can be seen that the time-dependent variations tend to be strongly suppressed with in increasing frequency in the chiral structure compared to the achiral one. In the chiral molecule, there is a clear trend of decreasing amplitude in the temporal response the higher the frequency, a trend which cannot be discerned in the achiral molecule.

In **Figure S10E** and **S10F** we also plot the associated time-dependent spin-polarization for the chiral and achiral molecules, respectively, and there are two striking differences between the induced spin-polarizations. First, while there is a significant dynamical spin-polarization in the chiral molecules, there is none at all in the achiral (notice that the vertical scale is in units of 10^{-15}). Second, for the chiral molecule, also the spin-polarization tends to become increasingly time-independent with increasing frequency, whereas there are hardly any changes in the achiral molecule.

The results from the calculations clearly point to that the electric and spin polarizations are intimately related in chiral molecules, while these quantities may be considered as independent in achiral. This conclusion can be drawn from three observations. First, since the densities of electron states in the two molecules are identical (**Figure S10B**), one can effectively exclude the possibility that the responses to the time-dependent perturbation would be a density effect. Second, since the chiral molecule develops a spin-polarization whereas the achiral does not, in response to the time-dependent perturbation, we can deduce that the induced spin-polarization is the key quantity that is distinct between the two molecules. Third, the fact that the spin-polarization in the chiral molecule becomes increasingly time-independent at a non-vanishing mean value with increasing frequency (**Figure S10E**), indicates that charges with different spins accumulate in different spatial locations and that there is a spatial imbalance built up through this differentiation of the spin. This spatial imbalance can, hence, be detected as an electric polarization (**Figure S10C**).

Materials and Methods

DNA and cloning. Synthetic gene fragments encoding P-loop prototypes were sourced from *Twist Biosciences* and cloned into a pET29(+)b expression vector using the standard restriction free cloning method. Similarly, mutant prototypes were generated using standard site-directed mutagenesis via restriction-free cloning with primers obtained from *Integrated DNA Technologies* (IDT).

Expression and purification of P-loop prototypes. All P-loop prototypes have a C-terminal Trp residue for concentration determination (the prototypes are otherwise devoid of aromatic residues) followed by a 6xHis tag for purification. DNA and amino acid sequences are provided in **Tables S1** and **S2**. Following purification, the yield and purity of purified proteins was assessed by SDS-PAGE. Typically, four peak elution fractions (~7.5 mL) were pooled together and subjected to two rounds of dialysis (2 h at room temperature followed by overnight dialysis at 4 °C) against buffer containing 50 mM Tris pH 8.0 and 100 mM NaCl. P-loop prototypes generally precipitate during the dialysis step and require an osmolyte such as L-arginine to be resolubilized. The samples were centrifuged and pellets containing the P-loop prototypes were resolubilized in buffer containing 50 mM Tris pH 8.0, 100 mM NaCl, and 1 M L-arginine ("solubilization buffer"). Aliquots of 100-200 µM P-loop prototypes in solubilization buffer were stored at 4 °C and remained soluble and active for at least 10-14 days.

Total peptide synthesis. The N-αβα prototype with either a C-terminal *L*-Cys (N-αβα_LcysEnd) or *D*-Cys (N-αβα_DcysEnd) residue were purchased from *Synpeptide*. Peptides were dissolved in 10% Acetonitrile + 90% Millipore water according to manufacturer's instructions to produce 2.5 mM stock solutions. HhH peptides were synthesized in house and their foldedness was assessed (**Supplementary Methods**, **Figures S1** and **S2**). Peptides were divided into aliquots and stored at -20 ºC until use.

Preparation of the ferromagnetic substrate. Ferromagnetic substrates were prepared as described previously. [28] An 8 nm Ti adhesive layer was deposited onto a p-type, boron-doped silicon wafer, ⟨100⟩ ± 0.9°. Next, a 100 nm nickel layer, which serves as the ferromagnetic layer, was deposited onto the adhesive-coated Si wafer using an electron beam evaporator. Finally, the Ni layer was coated with a 5 nm layer of gold. The evaporator chamber was kept under high

vacuum (<10^{-7} Torr) and ambient temperature during deposition of the metal layers. Substrates were cut into 23 mm × 23 mm squares using a diamond cutter. The substrate pieces were cleaned by boiling in acetone for 10 min, boiling in ethanol for 10 min, and sonicating in water for 1 min to dislodge any broken debris from the surfaces.

Drop casting. P-loop prototypes were diluted to 100 μM and dialyzed into 50 mM Tris pH 8.0. A ten-fold molar excess of TCEP was added to reduce the C-terminal cysteines. Samples were incubated at room temperature (for biologically purified proteins) or on ice (for synthetic peptides) for 3 hr to ensure complete reduction of all cysteine residues. To remove TCEP, samples were passed through a BioRad *Micro Bio-Spin* P-6 column equilibrated in 50 mM Tris pH 8.0. The protein concentration was measured by spectrophotometry and the final peptide concentration was adjusted to 80 μM. The HhH peptides were dissolved in 50 mM Tris buffer pH 7.5 and 150 mM NaCl. The concentration of peptide was then adjusted to 20 μM and verified using spectrophotometry. A five-fold molar excess of TCEP was added to reduce disulfide bonds. TCEP was removed as above.

Cleaned substrate (described above) were dried using an N_2 gas gun one at time and immediately drop cast by placing 30 μL of protein sample in the center of the substrate. The substrates with drop-casted proteins were placed in a Petri dish, sealed with a parafilm, and stored overnight (~16 hr) at room temperature (biologically produced proteins) or 4 °C (synthetic peptides).

Verification of monolayer formation from overnight incubation of proteins with Au-coated substrates was performed using polarization modulation-infrared reflection-adsorption spectroscopy (PM-IRRAS) and atomic force microscopy as described later **(Figures S3**, **S4** and **S5**). FM-substrates were washed three times with 1 mL of 50 mM Tris buffer pH 8.0. A small scratch at the center of each substrate was made using a diamond cutter. The scratch served as a reference point for microscopy experiments.

dsDNA hybridization. The dsDNA solutions were prepared by mixing the two complementary strands of ssDNA in 0.4 M phosphate buffer pH 7.2. Strands were denatured at 60 °C for 10 min and annealed by cooling to 15 °C at the rate of 1 °C/min. The annealed DNA was passed through a Micro Bio-Spin P-30 column equilibrated in 50 mM Tris pH 8.0 and concentration was measured

by spectrophotometry. Finally, DNA samples were adjusted to a concentration of 1 µM duplex DNA.

Microscopy measurements. Binding kinetics of ssDNA to P-loop prototypes or dsDNA to HhH peptides anchored to ferromagnetic (FM) substrates were monitored using a ZEISS Axio-Observer.Z1 inverted fluorescence widefield microscope. Prepared substrates were then placed on a permanent 0.42 T magnet with the North Pole facing the substrate and secured using adhesive tape. In parallel, 80 µL fluorescently labeled (6-FAM) ssDNA or dsDNA was placed at the center of a 35 mm glass bottom Petri dish (*MAKTEK)*, which was then transferred to the microscope stage. The magnet-substrate assembly was then inverted onto the Petri dish containing the fluorescent DNA solution.

Images were acquired using a 40× air objective lens (LD Plan-Neofluar 40x /0.6). Using a GFP filter, the emitted fluorescence was collected and separated from the excitation beam by two dichroic beam splitters along the optical path. The light was then routed to an avalanche photodiode (APD) for fluorescence imaging. Before initiating test experiments, a control experiment was carried out to focus the z-position at the surface-solution interface. Focusing was done by locating the scratch on the FM-substrate (described above). The z-position was then fixed for all the experiments. Experiments were initiated and fluorescence intensity images were acquired using the *Slidebook* software. Images were collected every 30 s for a total of 15 min. We selected the same 400-pixel x 400-pixel region of each image using the scratch as a reference point. The increase in fluorescence relative to initial fluorescence value (F/F_0) was calculated and plotted versus time. Each experiment was repeated three times and errors are reported as standard deviations. The entire procedure was then repeated with the South magnet pointing towards the substrate. The F/F_0 values were fitted to *the one phase association* equation (eq. 1) in GraphPad Prism software to estimate the rate constants.

$$Y = Y_0 + (Plateau - Y_0) \times (1 - exp(-k \times X)) \qquad \text{(eq. 1)}$$

Where Y_0 is the F/F_0 value at time zero, *Plateau* is the F/F_0 value at equilibrium, and k is the rate constant of the association process expressed in inverse time (s^{-1}) units.

Macroscopic contact potential difference (CPD) measurements. CPD measurements were performed using a commercial Kelvin probe (Delta Phi Besocke, Jülich, Germany) confined inside

a Faraday cage under atmospheric pressure. A general Kelvin probe set up includes a metallic probe electrode (gold grid) which is placed near the surface of the sample to form a capacitor. The distance between the probe electrode and the surface of the sample is periodically varied to create a frequency dependent capacitance. An AC voltage is generated across the gap and is proportional to the voltage difference between the sample and the probe electrode. Rather than measuring AC voltage directly, a DC voltage is generally applied to nullify the response, which measures the CPD. The CPD signal is allowed to stabilize before recording.

The substrates were p-doped silicon wafers Si ⟨100⟩ upon which Ti/Ni/Au 8nm/60nm/8nm with 0.3Å/sec, 0.5Å/sec, and 0.3Å/sec deposition rate at 10^{-8} Torr pressure were grown by an electron beam evaporator. After deposition, the substrates were cleaned with boiling acetone followed by boiling ethanol for 15 minutes each. Immediately after the cleaning, the substrates were dried under nitrogen flow. In parallel, the N-αβα_LcysEnd peptides were incubated with TCEP for reduction of the terminal cysteine as described above. TCEP was removed and the peptides were drop casted on the clean, dry substrates and incubated for 16 hours at 4 °C. Substrates were washed three times with 50 mM Tris pH 8.0, dried with nitrogen gas, and placed on a permanent magnet with either the North polarity facing towards (UP) or away from (DOWN) the substrate. A drop of TC-ssDNA was placed onto the substrates and incubated for 30 min. The dsDNA is then aspirated with a pipette, dried under nitrogen flow, and CPD measurements were performed.

Synthesis and purification of peptides with the HhH motif

Synthesis: As described in the ref. [3], *L*-Precursor and *D*-Precursor peptides, were synthesized on 2-Chlorotrityl-resin (loading 0.3 mmol/g, on a 0.25 mmol scale) on automated peptide synthesizer. Peptides were deprotected and cleaved to give 1356 mg of crude *L*-Precursor and 405 mg of crude *D*-Precursor.

Purification: 200 mg of crude *L*-Precursor and 100 mg of crude *D*-Precursor peptides were purified by RP-HPLC (XSelect C18 column, 5 µm, 130 Å, 30 × 250 mm) using a gradient of 30-60% B over 42 min to give pure *L*-Precursor (68 mg, 34% yield) and pure *D*-Precursor (26 mg, 26% yield). The HPLC analysis (**Figure S1**) was carried out on a C4 analytical column.

Circular Dichroism (CD) characterization of the HhH peptides

Secondary structural characteristics of *L*- and *D*-Precursor peptides was assessed by CD measurements. The CD spectroscopy measurements were performed by using a using a nitrogen

purged Chirascan™-Plus spectrometer, (Applied Photophysics, UK), with a thermoelectrically controlled single cell holder. The measurements were conducted at 1 s per point, 1 nm step size, and 1 nm bandwidth. The optical path of the quartz cuvette used was 0.1 cm. CD spectra of *L*- and *D*-Precursor indicates that the peptides are largely unfolded and are mirror images of each other (**Figure S2**)

Polarization modulation-infrared reflection-adsorption spectroscopy (PM-IRRAS) characterization of peptides.

PM-IRRAS was carried out to ensure overnight (16 hr.) incubation of FM-substrates with N-αβα prototype and HhH peptides results in a monolayer formation. For these experiments we used a 100 nm Au-coated silicon substrate. Spectrum was obtained for overnight adsorbed proteins by accumulating 2000 scans with the samples mounted at Brewster angle of incidence of 80 on a Nicolet 6700 FTIR with a PEM-90 photoelastic modulator. The spectra of peptide monolayers showed two characteristic peaks at 1670 and 1540 cm^{-1}, typical of amide-I (stretching mode of the CO bond) and amide-II (N−H in- plane bending mode and C−N stretching mode), respectively (**Figure S3** and **S4**).

Atomic Force Microscopy (AFM) for topography analysis.

AFM topography of N-αβα prototype and L-*Precursor* peptide monolayer on gold coated Si surface was scanned by Bruker-AFM MultiMode 8 in tapping mode with sharp silicon tips (radius = 7 nm, spring constant = 2 N/m, Model AC240TS-R3 from Oxford instruments) (**Figure S5**).

Simulation methodology

The simulations are based on the time-dependent Green function for the molecule in presence of a time-dependent disturbance. The molecule is modeled by the Hamiltonian $\mathcal{H} = \Psi^{\dagger}(H_0 + H_1)\Psi + \mathcal{H}_{ph}$, where $\Psi = \{\psi_m\}_{m=1}^{\mathbb{M}}$ is a column vector of the $\mathbb{M}$ spinors $\psi_m = (\psi_{m\uparrow}\psi_{m\downarrow})^t$, and H_0 and H_1 are the non-interacting and interacting contributions to the spectrum, while $\mathcal{H}_{ph} = \sum_{\mu}\omega_\mu b_\mu^{\dagger} b_\mu$ represents the collective nuclear vibrations distributed over the modes μ with energy ω_μ which are created and annihilated by the operators $b_\mu^{\dagger}$ and b_μ, respectively. Here, the first contribution to $\mathcal{H}$ can be written

$$H_0 = \left\{\epsilon_m \delta_{mn} + \sum_{s=\pm1}(-t_0\delta_{nm+s} + i\lambda_0 \boldsymbol{v}_m^{(s)} \cdot \boldsymbol{\sigma}\delta_{nm+2s})\right\}_{mn=1}^{\mathbb{M}},$$

where ϵ_m denotes the ons-site HOMO level, t_0 is the nearest-neighbor hopping, and $i\lambda\boldsymbol{v}_m^{(s)} \cdot \boldsymbol{\sigma}$ is the next-nearest neighbor hopping. The latter component connects the chirality $\boldsymbol{v}_m^{(s)}$ with the spin-orbit interaction $i\lambda_0$. The second contribution to $\mathcal{H}$ is given by

$$H_1 = \left\{\sum_{s=\pm1}(-t_1\delta_{nm+s} + i\lambda_1 \boldsymbol{v}_m^{(s)} \cdot \boldsymbol{\sigma}\delta_{nm+2s})\sum_{\mu}(b_\mu + b_\mu^{\dagger})\right\}_{mn},$$

where t_1 and λ_1 define corresponding vibration assisted nearest and next-nearest neighbor interactions, through the coupling to the nuclear displacement operator $\sum_{\mu}(b_\mu + b_\mu^{\dagger})$.

The molecule is coupled to a time-independent external charge $\psi_0^{\dagger}\psi_0$ via the charge-charge interaction $V(t)\psi_0^{\dagger}\psi_0\psi_1^{\dagger}\psi_1$, with the time-dependent coupling parameter $V(t) = U_0(1 + cos\hbar v_0 t)$, where v_0 defines the frequency of the coupling.

The molecular charge and spin distributions are calculated using non-equilibrium Green functions, $\mathbb{G}(t,t') = \{\boldsymbol{G}_{mn}(t,t')\}_{mn}$, where each matrix element $\boldsymbol{G}_{mn}(t,t') = (-i)\langle T\psi_m(t)\psi_n^{\dagger}(t')\rangle$ is a

2×2-matrix propagator. The site resolved time-dependent charge and spin densities are given by $\langle n_m(t)\rangle = (-i)sp\boldsymbol{G}^{<}_{mm}(t,t)/2$ and $\langle \boldsymbol{s}_m(t)\rangle = (-i)sp\boldsymbol{\sigma}\boldsymbol{G}^{<}_{mm}(t,t)/4$, respectively. The equation of motion for $\mathbb{G}(t,t')$ is approximated by

$$(i\partial_t - H_0)\mathbb{G}(t,t') = \delta(t-t') + \langle\psi_0^\dagger\psi_0\rangle(t)\mathbb{V}(t)\mathbb{G}(t,t') + \int \boldsymbol{\Sigma}(t,\tau)\mathbb{G}(\tau,t')d\tau,$$

where $\mathbb{V}(t)$ is the matrix coupling the external charge $\langle\psi_0^\dagger\psi_0\rangle(t)$ to $\boldsymbol{G}_{11}(t,t^{'})$ and $\boldsymbol{\Sigma}$ is the self-energy caused by the interactions with the nuclear vibrations.

Here, we define the Green function $\mathbb{G}_0$ to include the interactions between electrons and nuclear vibrations, that is,

$$(i\partial_t - H_0)\mathbb{G}_0(t,t') = \delta(t-t') + \int \boldsymbol{\Sigma}(t,\tau)\mathbb{G}(\tau,t')d\tau,$$

which is time-independent. Hence, we may write

$$\mathbb{G}_0(z) = (z - H_0 - \boldsymbol{\Sigma}(z))^{-1}.$$

Using this formulation, the time-dependent Green function is provided by the expression

$$\mathbb{G}(t,t') = \mathbb{G}_0(t,t') + \int \mathbb{G}_0(t,\tau)\mathbb{V}_0(\tau)\mathbb{G}(\tau,t')d\tau,$$

where $\mathbb{V}_0(t) = \langle\psi_0^\dagger\psi_0\rangle(t)\mathbb{V}(t)$. For computational purposes, this expression is approximated by

$$\mathbb{G}(t,t') \approx \mathbb{G}_0(t,t') + \int \mathbb{G}_0(t,\tau)\mathbb{V}_0(\tau)\mathbb{G}_0(\tau,t')d\tau.$$

The lesser Green function $\mathbb{G}^{<}(t,t^{'})$ is, then, given by the expression

$$\mathbb{G}^{<}(t,t^{'}) \approx \mathbb{G}_0^{<}(t,t^{'}) + \int(\mathbb{G}_0^{r}(t,\tau)\mathbb{V}_0(\tau)\mathbb{G}_0^{<}(\tau,t^{'}) + \mathbb{G}_0^{<}(t,\tau)\mathbb{V}_0(\tau)\mathbb{G}_0^{a}(\tau,t^{'}))d\tau.$$

Supplementary Figures

A.

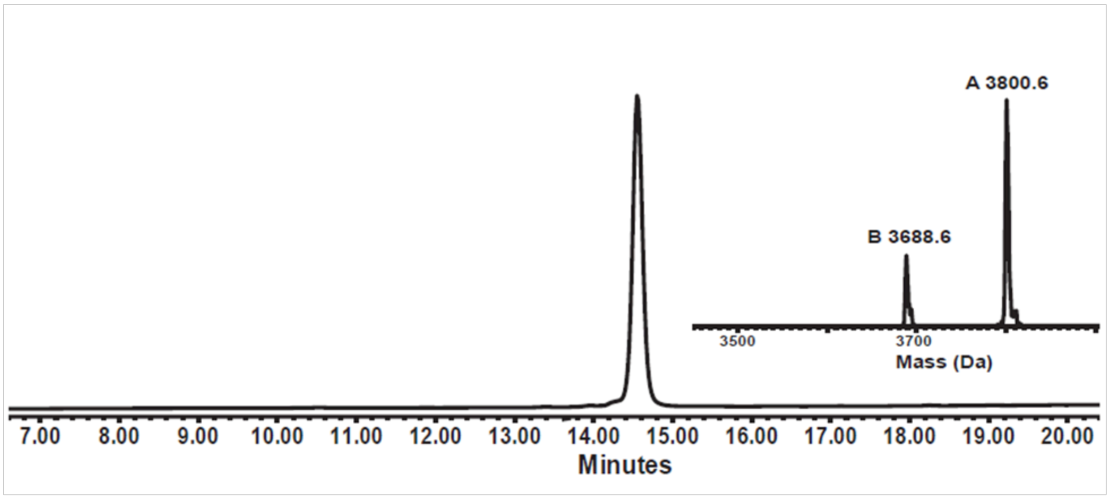

B.

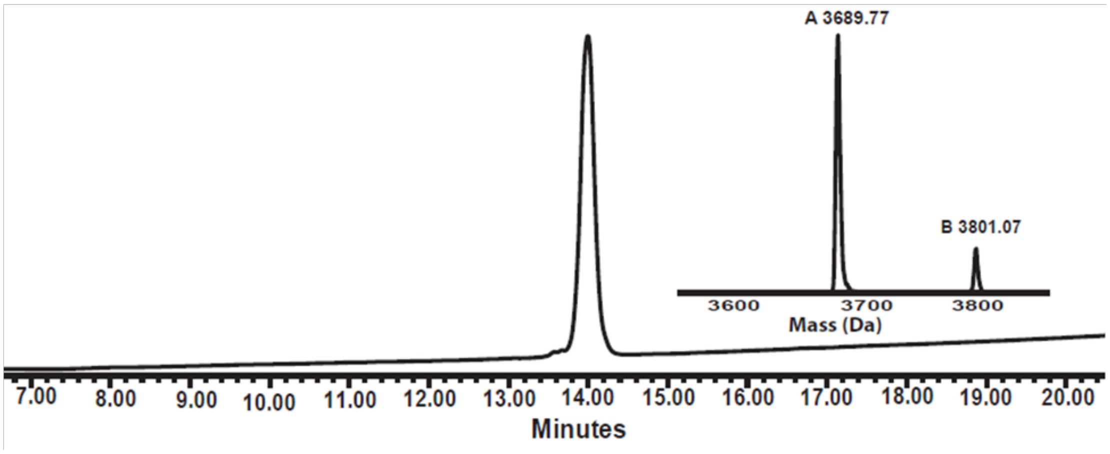

Figure S1: HPLC chromatograms and ESI-MS for **A.** purified *L*-Precursor, with the inset showing the corresponding mass (calc. 3689.3 Da; obs. 3688.6 Da, [M+TFA] 3800.6 Da) and **B.** purified *D*-Precursor, with the inset showing the corresponding mass (calc. 3689.35 Da; obs. 3689.77 Da, [M+TFA] 3801.07 Da).

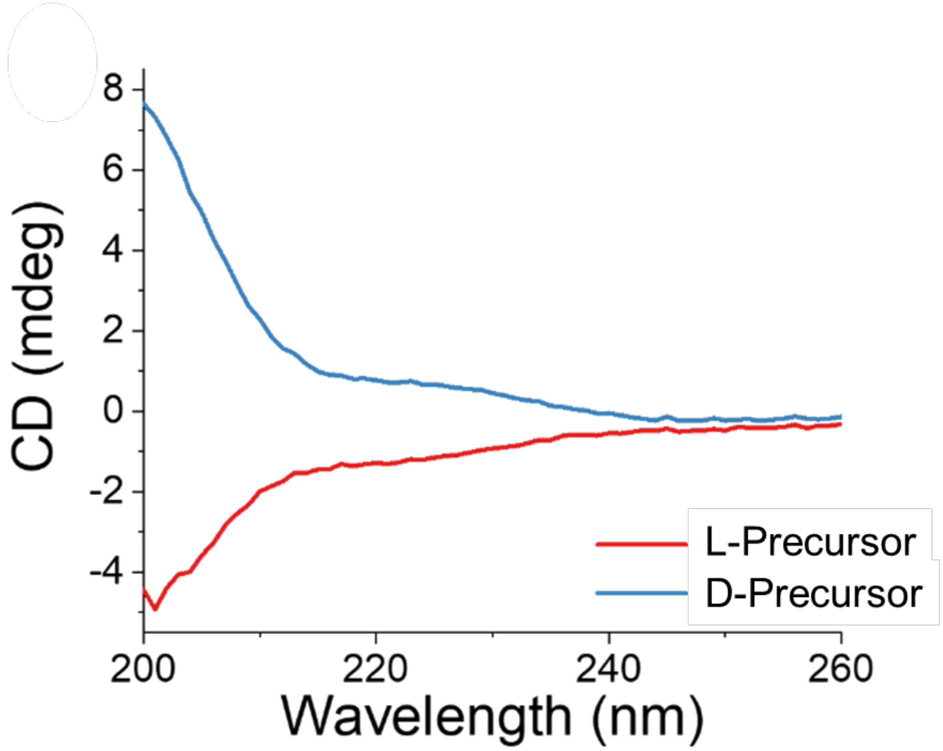

Figure S2: CD spectra of *L*- and *D*-Precursor peptides in 50 mM Tris buffer with 150 mM NaCl showing a predominantly unfolded conformation.

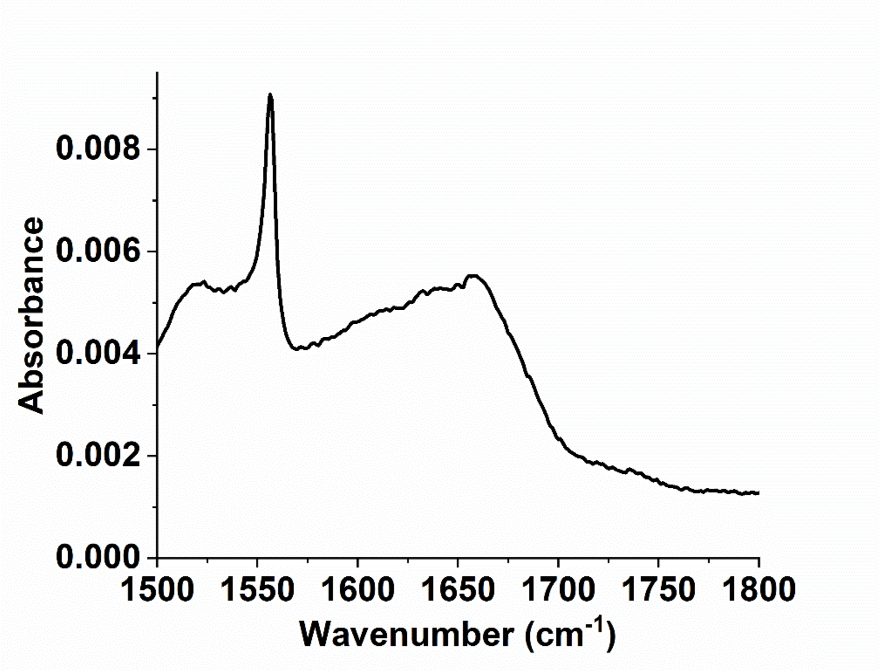

Figure S3: A representative PM-IRRAS spectra of the N-αβα prototype incubated for 16 hr. on Au substrate. Here 80 µM peptide was used to grow the monolayer. The peak around 1670 and 1540 cm^{-1} correspond to the characteristic C=O stretching N-H in plane bending mode and C−N stretching mode vibrations, commonly named as amide-I and amide-II peak.

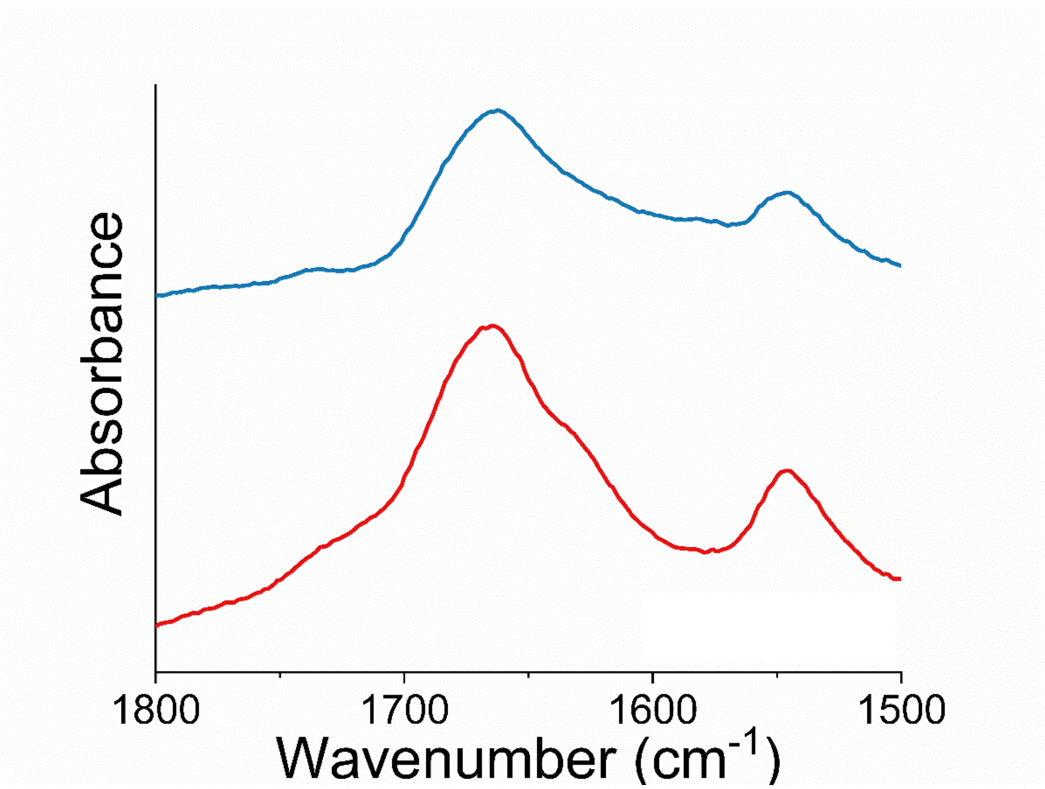

Figure S4: PMIRRAS spectra of *L*-Precursor (red) and *D*-Precursor (blue) peptide incubated for 16 h on Au substrate. Here 20 µM peptide was used to grow the monolayer. The peak around 1670 and 1540 cm^{-1} correspond to the characteristic C=O stretching N-H in plane bending mode and C−N stretching mode vibrations, commonly named as amide-I and amide-II peak.

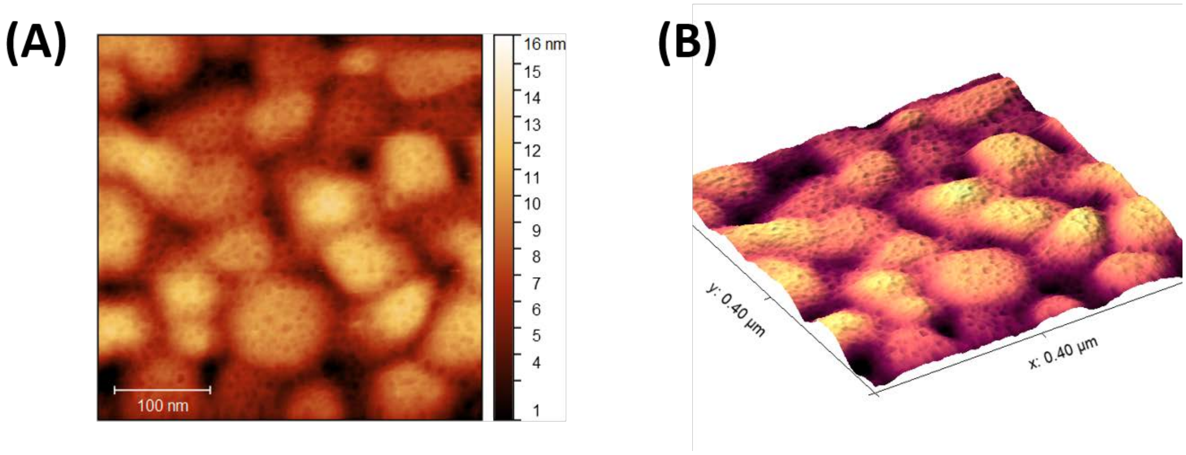

Figure S5: A representative AFM topographic **A**. 2D and **B.** 3D image of the *L*-Precursor HhH peptide monolayer on Au surface.

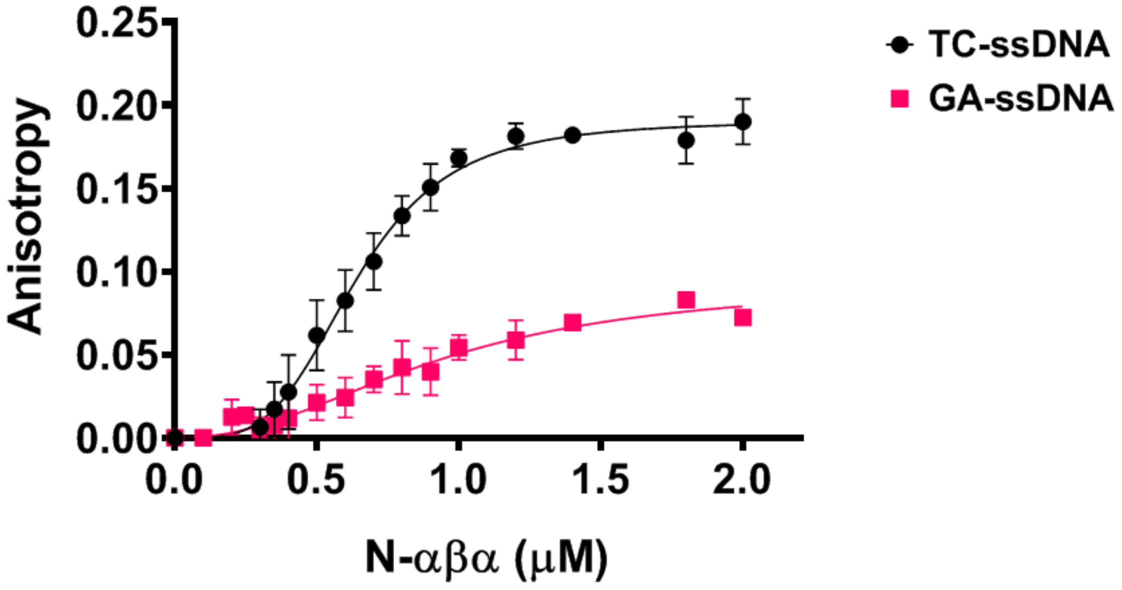

Figure S6: Binding of N-αβα prototype to DNA measured by fluorescence anisotropy**.** Shown is the normalized anisotropy signal. Increase in anisotropy indicates binding. Figure is reproduced from ref. [4]. Shown here is fluorescence anisotropy plot of varying concentrations of the N-αβα prototype with TC- and GA-ssDNA oligos (**Supplementary Table S1**). Shown are average values from four to eight independent experiments with vertical error bars representing the SD values. For details on methods see ref. [4]. The KD values are listed in table S4.

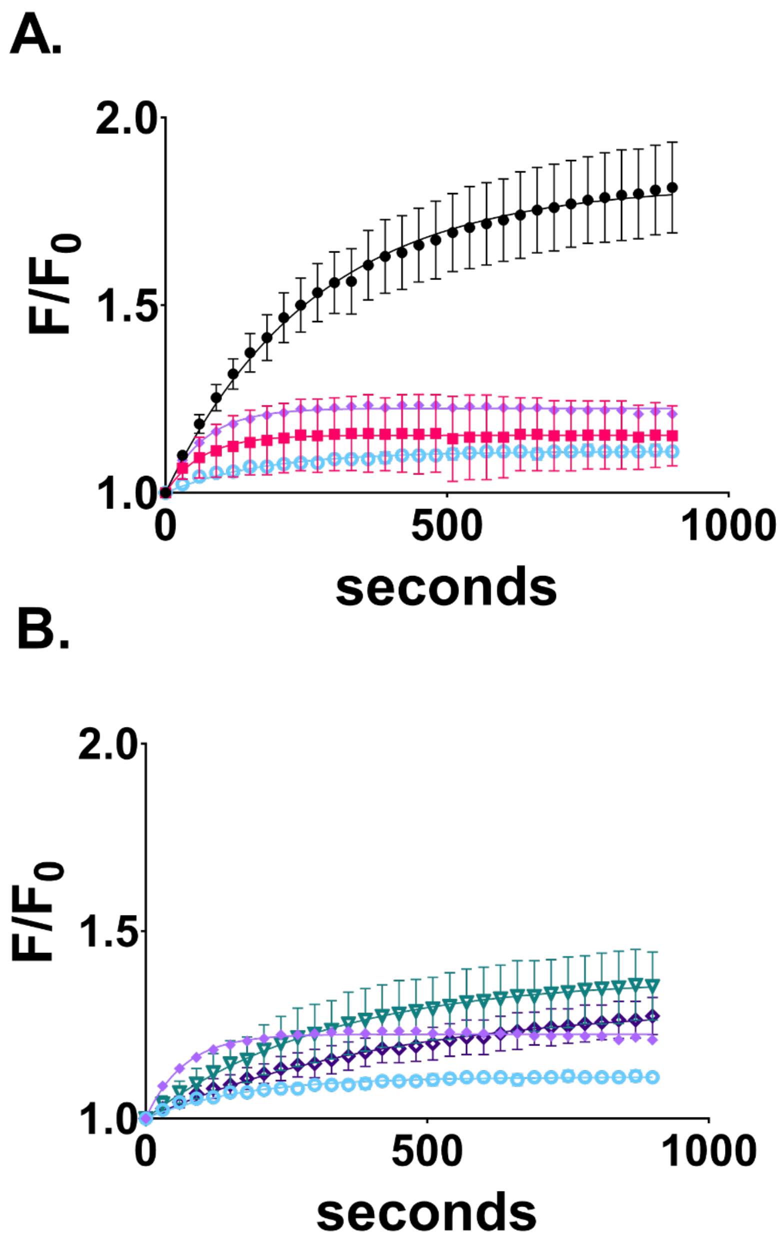

Figure S7: Comparative adsorption profiles of N-αβα prototype to DNA and fluorescein dye. A. Adsorption isotherms of N-αβα binding to TC-ssDNA and fluorescein dye for UP and DOWN orientations of the magnet. *TC-ssDNA + UP (black)*, *TC-ssDNA + DOWN (pink)*, *fluorescein + UP (purple)*, *fluorescein + DOWN (blue)* **B.** Adsorption isotherms of N-αβα binding to GA-ssDNA and fluorescein dye for UP and DOWN orientations of the magnet. *GA-ssDNA + UP (green)*, *GA-ssDNA + DOWN (violet)*, *fluorescein + UP (purple)*, *fluorescein + DOWN (blue)* Data were plotted as F/F_0 vs seconds and fitted to standard one-phase association equation using GraphPad Prism software. Error bars represent standard deviation from three to four independent measurements. Adsorption curves for TC-ssDNA and GA-ssDNA are same as in figure 2 of main text.

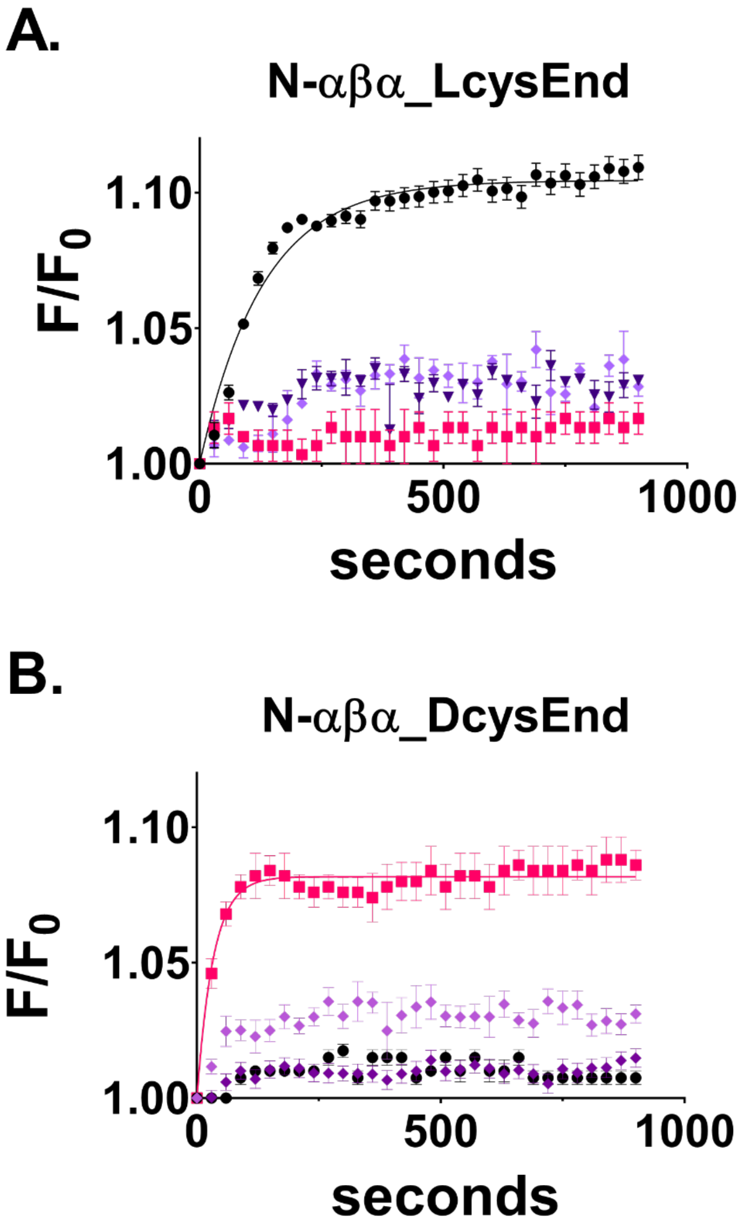

Figure S8: Comparative adsorption profiles of synthetic N-αβα prototypes to TC-ssDNA and fluorescein dye. A. Adsorption isotherms of N-αβα_LcysEnd and **B.** N-αβα_DcysEnd binding to TC-ssDNA and fluorescein dye for UP and DOWN orientations of the magnet. *TC-ssDNA + UP (black)*, *TC-ssDNA + DOWN (pink)*, *fluorescein + UP (purple)*, *fluorescein + DOWN (violet)*. Data were plotted as F/F_0 vs seconds and fitted to standard one-phase association equation using GraphPad Prism software. Error bars represent standard deviation from three to four independent measurements. Adsorption curves for N-αβα_LcysEnd and N-αβα_DcysEnd are same as in figure 3 of main text.

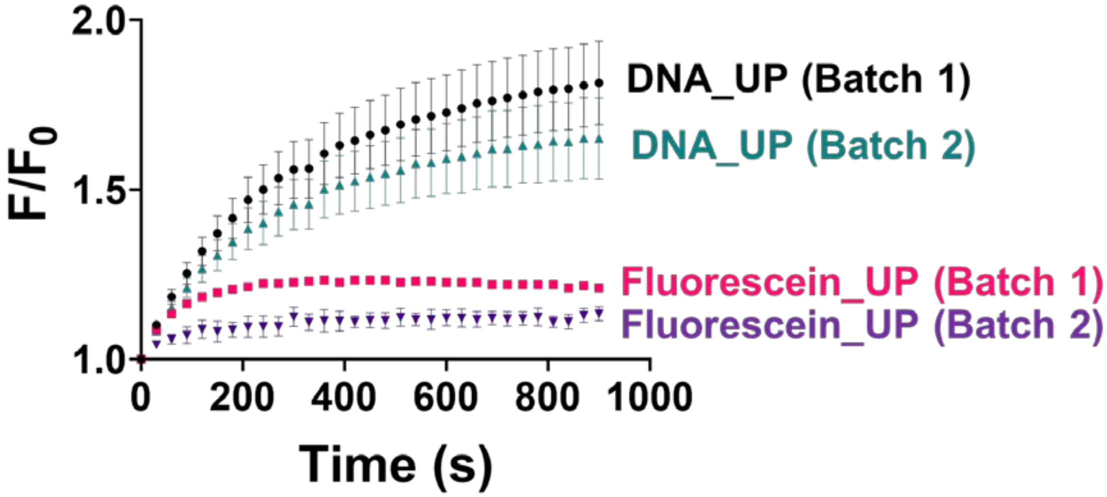

Figure S9: Binding of TC-ssDNA to N-αβα prototype for two independent batches of protein purification. Shown here are binding curves of TC-ssDNA and fluorescein (i.e., digested DNA) to adsorbed N-αβα prototype for UP orientation, as in Figure 2 of main text, for two independent batches of purification rounds. Batch 1 traces are same as in Figure 2A of main text.

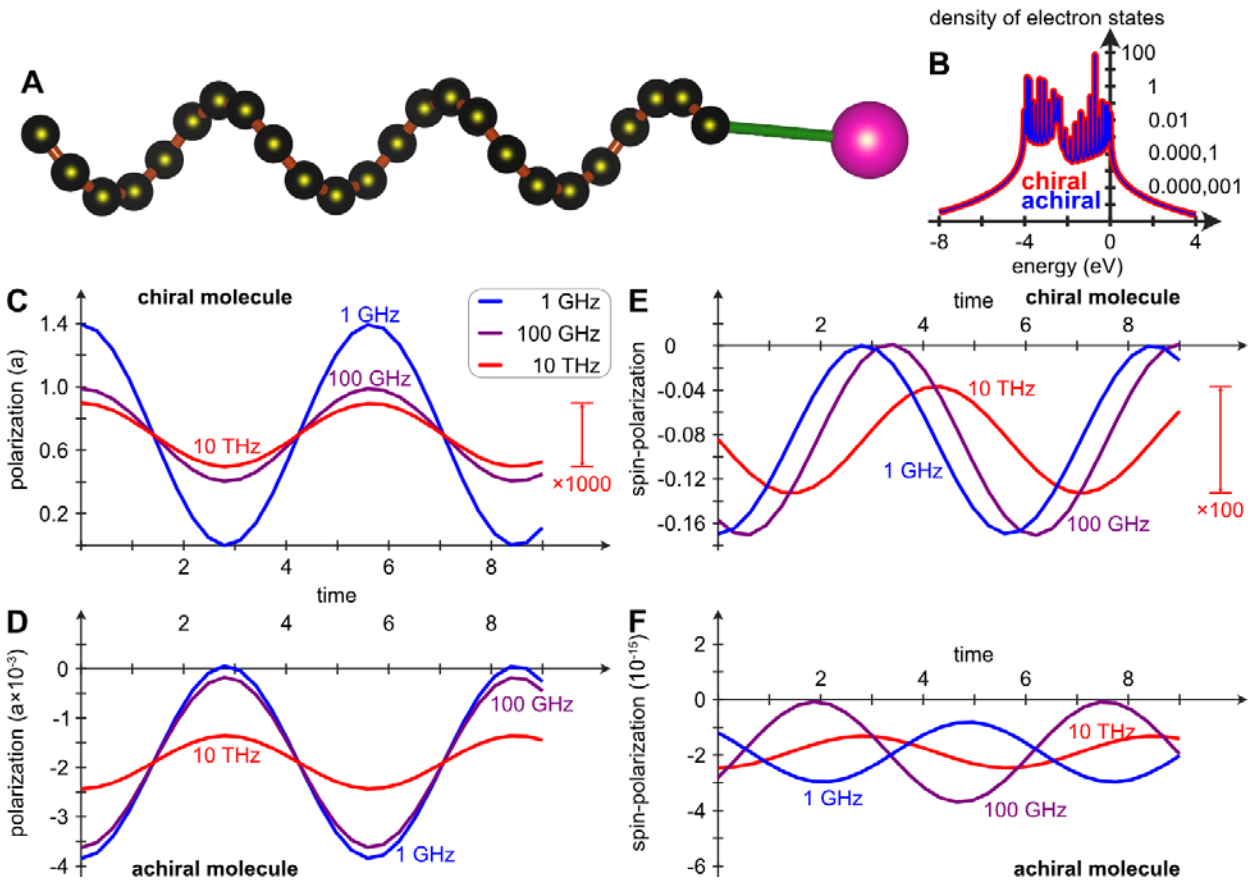

Figure S10: A. Schematic of the chiral system (black balls, brown bonds) connected to the external charge (magenta ball, green coupling). The corresponding achiral system is represented by a zig-zag chain of sites. **B**. Density of electron states for the chiral (red) and achiral (blue) molecules. The chemical potential is set to 0. **C**. & **D**. Time-dependent variations of the dominant component of the electric polarization vector $\boldsymbol{P}$ for the **C**. chiral and **D**. achiral system, under the time-dependent coupling between the system and external charge. The time-dependent coupling varies harmonically with frequencies $\nu_0 = 1$ GHz, 100 GHz, 10 THz. Results with more frequencies are presented in the supplementary information. **E**. & **F**. Time-dependent variations of the spin-polarizations corresponding to the polarizations in **C**. & **D**. The sites in the system host electron levels at $\epsilon_m = -2t_0$ and are elastically coupled by the nearest neighbor coupling $t_0 = 1$ eV and next-nearest neighbor coupling $\lambda_0 = 1$ meV, whereas the inelastic coupling strengths are $t_1 = t_0/10$ and $\lambda_1 = \lambda_0/10$. The system vibrates with the frequency corresponding to the energy $\omega_0 = 0.5$ eV, the helix radius a $= 5$ Å, and the temperature is set to $T = 300$ K.

Table S1: Coding DNA sequences of P-loop prototypes used in this study.

Protein	Coding DNA sequence
N-αβα (ref. [4])	ATGACTCGTGATGATGCAAAACGTGTAGCGGAAGAAGCGA AGCGTCGCGGTGTTGGTAGCGGCCGTGTGATTATCGTAATT GTGGGTCCAAGCGGCGCAGGCAAAACCACCCTGCTCGAAC TGGCTAAAGAAGCTAAGAAGGAGGTGTGGCTCGAGCACCA CCACCACCATCAC**TGA**
N-αβα with terminal cysteine (this study)	ATGACTCGTGATGATGCAAAACGTGTAGCGGAAGAAGCGA AGCGTCGCGGTGTTGGTAGCGGCCGTGTGATTATCGTAATT GTGGGTCCAAGCGGCGCAGGCAAAACCACCCTGCTCGAAC TGGCTAAAGAAGCTAAGAAGGAGGTGTGGCTCGAGCACCA CCACCACCATCAC**TGCTGA**
N-βα (ref. [4])	ATGCGTGTGATTATCGTAATTGTGGGTCCAAGCGGCGCAGG CAAAACCACCCTGCTCGAACTGGCTAAAGAAGCTAAGAAG GAGGTGTGGCTCGAGCACCACCACCACCATCAC**TGA**
N-βα with terminal cysteine (this study)	ATGCGTGTGATTATCGTAATTGTGGGTCCAAGCGGCGCAGG CAAAACCACCCTGCTCGAACTGGCTAAAGAAGCTAAGAAG GAGGTGTGGCTCGAGCACCACCACCACCATCAC**TGCTGA**

The DNA sequences of N-αβα and N-βα prototype are shown from reference [4]. The DNA sequence codon for the incorporated cysteine is shown in bold red. The terminal stop codon is shown in bold.

Table S2: Amino acid sequences of protein constructs used in this study.

Prototype	Amino acid sequence	Molecular weight (kDa)
**Prototypes expressed in purified from BL.21 E. coli cells**		
N-αβα (ref. [4])	MTRDDAKRVAEEAKRRGVGSGRVIIVIV**GPSG AGKT**TLLELAKEAKKEV*WLEHHHHHH*	6.4
N-αβα with terminal cysteine	MTRDDAKRVAEEAKRRGVGSGRVIIVIV**GPSG AGKT**TLLELAKEAKKEV*WLEHHHHHH***C**	6.5
N-βα (ref. [4])	MRVIIVIV**GPSGAGKT**TLLELAKEAKKEV*WLE HHHHHH*	4.3
N-βα with terminal cysteine	MRVIIVIV**GPSGAGKT**TLLELAKEAKKEV*WLE HHHHHH***C**	4.4
**Chemically synthesized constructs**		
N-αβα_LcysEnd	MTRDDAKRVAEEAKRRGVGSGRVIIVIV**GPSG AGKT**TLLELAKEAKKEV**C**	5.3
N-αβα_DcysEnd	MTRDDAKRVAEEAKRRGVGSGRVIIVIV**GPSG AGKT**TLLELAKEAKKEV**C**	5.3
L-Precursor	CSIERIRRASVEELTEV**PGIGP**RLARRILERL	3.7
D-Precursor	CSIERIRRASVEELTEV**PGIGP**RLARRILERL	3.7

Bacterially expressed and purified prototypes contained a C-terminal expression tag that included a tryptophan (W) residue to allow determination of protein concentration by absorbance at 280 nm, and a 6xHis tag for purification (annotated in italics). Terminal cysteine residues are shown in bold red. For synthetic N-αβα_DcysEnd, the terminal cysteine which is in the D-form is shown in underlined red. P-loop motif is shown in bold in all prototypes. For synthetic HhH peptides, the conserved PGIGP motif is shown in bold. The HhH peptide with *D*-form amino acids is shown in red. Molecular weight for each construct was calculated from the amino-acid sequence using ExPASy ProtParam tool [5]

Table S3: DNA oligonucleotides used in this study

Oligos	Sequence (5' to 3')	Figure panels from main text
TC-ssDNA	6-FAM - TACTTCTCTTCTCTCTCCTCGACT	2A, 2C, 3A, 3B, 4B
GA-ssDNA	6-FAM - AGTCGAGGAGAGAGAAGAGAAGTA	2B, 2C
DNA_{12}-sense	6-FAM - TAGATCGATCGC	3D, 3E
DNA_{12}-antisense	GCGATCGATCTA	3D, 3E

6-FAM = 6-Carboxyfluorescein

Table S4: Binding properties of N-αβα prototype to ssDNA constructs as measured by fluorescence anisotropy.

**ssDNA constructs**	**K_D (μM)**	**h**	**R^2**
TC-ssDNA	0.67 (±0.16)	3.92 (±0.64)	0.95
GA-ssDNA	1.70 (±0.68)	2.98 (±2.44)	0.83

Data is reproduced from ref. [4]. Values in parenthesis represents standard deviation from four to eight independent experiments.

REFERENCES

1. Fransson, J. Vibrational origin of exchange splitting and chiral-induced spin selectivity. *Phys. Rev. B* **102**, 235416 (2020).
2. Fransson, J. Charge and Spin Dynamics and Enantioselectivity in Chiral Molecules. *J. Phys. Chem. Lett.* **13**, 808–814 (2022).
3. Weil-Ktorza, O. *et al.* Functional Ambidexterity of an Ancient Nucleic Acid-Binding Domain. *bioRxiv* 2023.03.06.531422 (2023) doi:10.1101/2023.03.06.531422.
4. Vyas, P. *et al.* Helicase-Like Functions in Phosphate Loop Containing Beta-Alpha Polypeptides. *Proc. Natl. Acad. Sci.* **118**, (2021).
5. Gasteiger, E. *et al.* Protein identification and analysis tools on the ExPASy server. in *The proteomics protocols handbook* 571–607 (Springer, 2005).
